# Delivery of Hairpin RNAs and Small RNAs Into Woody and Herbaceous Plants by Trunk Injection and Petiole Absorption

**DOI:** 10.3389/fpls.2018.01253

**Published:** 2018-08-24

**Authors:** Athanasios Dalakouras, Wolfgang Jarausch, Guenther Buchholz, Alexandra Bassler, Mario Braun, Thorsten Manthey, Gabi Krczal, Michael Wassenegger

**Affiliations:** ^1^RLP AgroScience GmbH, AlPlanta - Institute for Plant Research, Neustadt, Germany; ^2^Institute of Plant Breeding and Genetic Resources ELGO-DEMETER, Thessaloniki, Greece; ^3^Centre for Organismal Studies Heidelberg, Heidelberg, Germany

**Keywords:** RNAi, dsRNA, siRNAs, trunk, petiole, *Malus domestica*, *Vitis vinifera*, *Nicotiana benthamiana*

## Abstract

Since its discovery, RNA interference has been widely used in crop protection. Recently, transgene-free procedures that were based on exogenous application of RNA molecules having the capacity to trigger RNAi *in planta* have been reported. Yet, efficient delivery of such RNA molecules to plants and particularly to trees poses major technical challenges. Here, we describe simple methods for efficient delivery of hairpin RNAs (hpRNAs) and small interfering RNAs (siRNAs) to *Malus domestica*, *Vitis vinifera*, and *Nicotiana benthamiana* that are based on trunk injection and/or petiole absorption. The applied RNA molecules were efficiently taken up and systemically transported. In apical leaves, the RNA was already detectable 1 day post-application (dpa) and could be detected at least up to 10 dpa, depending on the method of application. Confocal microscopy revealed that the uptaken and systemically transported RNA molecules were strictly restricted to the xylem and apoplast which may illustrate why the applied hpRNAs were not processed into siRNAs by plant DICER-LIKE (DCL) endonucleases. These innovative methods may have great impact in pest management against chewing and/or xylem sap-feeding vectors and eukaryotic pathogens that reside in the xylem.

## Introduction

In plants, RNA interference (RNAi) is triggered by double stranded RNAs (dsRNAs) which are processed by DICER-LIKE endonucleases (DCLs) into 21–24 nucleotide (nt) small interfering RNAs (siRNAs). SiRNAs are incorporated into the RNA-induced silencing complex (RISC) that contains an ARGONAUTE (AGO) protein. In general, RISC-loaded 21-nt siRNAs recognize by Watson:Crick hybridization complementary single stranded RNA transcripts which are then cleaved by RISC (Baulcombe, 1996; Hamilton and Baulcombe, 1999; Vaucheret, 2008; Voinnet, 2008). Importantly, RNAi is not cell autonomous in plants. Thus, siRNAs are transported through plasmodesmata into neighboring cells, and through the vasculature system to distant parts of the plant (Voinnet and Baulcombe, 1997; Yoo et al., 2004; Molnar et al., 2010; Melnyk et al., 2011a,b).

Although RNAi regulates normal plant development and genome stability, its role is also instrumental in the defense against invading nucleic acids and hostile organisms. Thus, since its breakthrough discovery 20 years ago by Fire et al. (1998), RNAi has been extensively used in crop improvement and protection platforms (Eamens et al., 2008; Martinez de Alba et al., 2013). So far, conventional RNAi applications have been mainly based on the use of transgenes and/or viral vectors that enabled direct production of dsRNA molecules (Baulcombe, 1996; Palauqui et al., 1997; Palauqui and Vaucheret, 1998; Ruiz et al., 1998; Hamilton and Baulcombe, 1999; Mette et al., 1999; Dalmay et al., 2000, 2001; Mette et al., 2000; Tenllado and Diaz-Ruiz, 2001). Yet, transgenic plants fall under the regulation of genetically modified organisms (GMOs) and their use has raised significant public and political concerns. Thus, the need for new sustainable, GMO-free and effective agricultural solutions comprising methods enabling the activation of RNAi through delivery of exogenous RNA molecules has emerged.

Bacterially expressed dsRNAs were shown to confer resistance in *Nicotiana benthamiana*, against *Pepper mild mottle virus* (PMMoV) (Tenllado et al., 2003), in *Zea mays*, against *Sugarcane mosaic virus* (SCMV) (Gan et al., 2010), in tobacco, against *Tobacco mosaic virus* (TMV) (Yin et al., 2010) and *Cucumber mosaic virus* (CMV) (Mitter et al., 2017) and in cucurbits, against *Zucchini yellow mosaic virus* (ZYMV) (Kaldis et al., 2017). In addition, we have previously demonstrated that high pressure spraying of *in vitro* synthesized siRNA molecules efficiently triggered local and systemic RNAi of a GREEN FLUORESCENT PROTEIN (GFP) transgene in *N. benthamiana* (Dalakouras et al., 2016). Moreover, *in vitro* transcribed dsRNAs conferred resistance in barley, against *Fusarium graminae* (Koch et al., 2016) and in tomato, against *Botrytis cinerea* (Wang et al., 2016). Yet, procedures exhibiting great agronomic and economic importance may involve pest management by RNAi-mediated targeting of insects and fungi. A plethora of RNAi-based assays with variable degrees of success have been developed in which insects are soaked in, injected with or fed with dsRNA solutions (Price and Gatehouse, 2008; Scott et al., 2013; Joga et al., 2016; Ghosh et al., 2017). In terms of field-scale applications, the challenging approach would be the delivery of RNA molecules to field test plants and monitoring of RNAi establishment in insects feeding on tissues and/or sap of these plants. Indeed, such approaches revealed promising results when dsRNA was exogenously delivered to Arabidopsis (to target stem-borer) (Li et al., 2015), to potato (to target *Colorado potato beetle*) (San Miguel and Scott, 2016), and to tomato (to target *Tuta absoluta* and *Diabrotica* spp.) (Ivashuta et al., 2015; Camargo et al., 2016).

Nevertheless, all exogenous RNA delivery methods that have been described above, referred to herbaceous plants. Woody plants are far more recalcitrant to such procedure, which may be also indicated by the scarcity of relevant reports. Yet, in a pioneering work, Hunter et al. (2012) showed that when 2.5 m tall citrus trees were exposed to *in vitro* transcribed dsRNA by root drenching and trunk injections, dsRNA was transported to the apical parts of the plant. However, dsRNA detection was only achieved by PCR-based methods, known to be extremely sensitive and also prone to false positives. Moreover, no information about the accumulation and processing of the applied dsRNA was provided. Here, we present novel and simple methods for efficient (as detected by Northern blot analysis) RNA delivery (hpRNAs, siRNAs) to *Malus domestica*, *Vitis vinifera* and *N. benthamiana*. The details of the application methods, the processing of the applied RNAs, their localization and function, as well as the implications of our findings in crop protection are discussed.

## Results and Discussion

### Delivery of hpRNA by Trunk Drilling and Injection

RNA molecules were delivered to two agronomical important woody plants, *M. domestica* and *V. vinifera*. Several *M. domestica* and *V. vinifera* endogenous micro RNAs (miRNAs) have been identified (Belli Kullan et al., 2015; Guo et al., 2016). Moreover, transgenic *M. domestica* and *V. vinifera* expressing dsRNAs were shown to efficiently trigger RNAi (Gilissen et al., 2005; Gambino et al., 2009). In addition, methods for virus-induced gene silencing (VIGS) were established for apple species (Kurth et al., 2012; Yamagishi and Yoshikawa, 2013). Thus, exogenous delivery of RNA molecules designed to trigger RNAi could serve as a means to suppress pests and pathogens in both of these woody plants.

**FIGURE 1:**
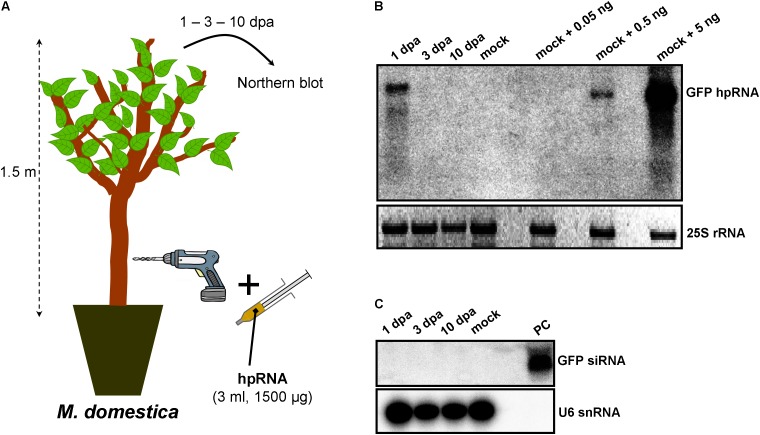
Delivery of exogenous hpRNA (500 nt) in apple by trunk drilling. **(A)** Apical leaves were harvested 1, 3, and 10 dpa and total RNA was extracted. **(B)** Northern blot for the detection of GFP hpRNA. Total RNA (5 μg) was analyzed in a 1.2% agarose/formaldehyde gel and the full-length GFP cDNA fragment (792 bp) was used as a hybridization probe. Mock RNA (from non-treated *M. domestica* plants) was spiked with dilutions of 5, 0.5, and 0.05 ng input hpRNA to demonstrate that in systemic leaves, the detected hpRNA is of the same size as the input hpRNA. The ethidium bromide stained gel depicts the 25S rRNA band and serves as an RNA loading control. **(C)** Northern blot for the detection of GFP siRNAs. Total RNA (20 μg) was analyzed in a 15% polyacrylamide gel and the full-length GFP cDNA fragment (792 bp) was used as a hybridization probe. As a positive control (PC) *in vitro* synthesized 22-nt GFP siRNAs (100 ng) were included. Hybridization of the membrane with a DNA oligo detecting the U6 snRNA served as an RNA loading control.

**FIGURE 2:**
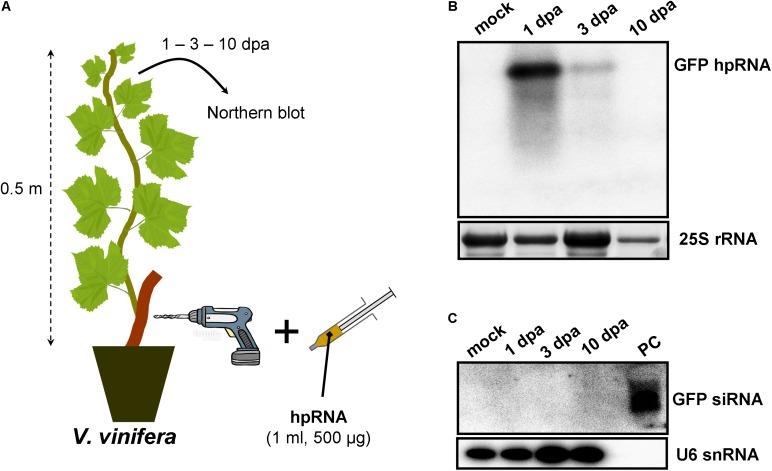
Delivery of exogenous hpRNA (500 nt) in *V. vinifera* by trunk drilling. **(A)** Apical leaves were harvested 1, 3, and 10 dpa and total RNA was extracted. **(B)** Northern blot for the detection of GFP hpRNA. Total RNA (5 μg) was analyzed in a 1.2% agarose/formaldehyde gel and the full-length GFP cDNA fragment (792 bp) was used as a hybridization probe. The ethidium bromide stained gel depicts the 25S rRNA band and serves as an RNA loading control. **(C)** Northern blot for the detection of GFP siRNAs. Total RNA (20 μg) was analyzed in a 15% polyacrylamide gel and the full-length GFP cDNA fragment (792 bp) was used as a hybridization probe. As positive control (PC) *in vitro* synthesized 22-nt GFP siRNAs (100 ng) were included. Hybridization of the membrane with a DNA oligo detecting the U6 snRNA served as an RNA loading control.

As RNA input, a 500-nt *in vitro* transcribed GFP hpRNA was used. The first method of RNA delivery involved drilling of the trunk of *M. domestica* (**Figure 1A**) and *V. vinifera* (**Figure 2A**). It has to be noted here that in the case of *V. vinifera*, since it is propagated by grafting, the hole was drilled into the woody rootstock (**Figure 2A**). Through these holes, the hpRNA was gently applied with the help of a 1 ml insulin syringe (without needle). The plants were transferred back to the glasshouse and kept at 25°C, 16/8 h light/dark. After this single hpRNA application, pooled leaves (distant from the site of application but not necessarily confined to young expanding leaves) from the upper parts of each plant were collected 1, 3, and 10 days post-application (dpa). Thus, from these leaves total RNA was extracted and subjected to Northern blot analysis. The data obtained for both, *M. domestica* and *V. vinifera* revealed high accumulation of hpRNA already 1 dpa but decreased at 3 and 10 dpa. (**Figures 1B, 2B**). Thus, the trunk drilling method resulted in the rapid uptake and systemic transport of the exogenous RNA, but the overall effect was not lasting more than 3 or 10 days (at least at a Northern blot analysis scale). To be noted, this transport was not affected by the grafting junction in *V. vinifera*, reminiscent of previous reports where the mobility of RNAi signals was not inhibited by graft junctions (Palauqui et al., 1996, 1997; Palauqui and Vaucheret, 1998; Palauqui and Balzergue, 1999; Mallory et al., 2003; Molnar et al., 2010). Importantly, Northern blot analyses revealed the accumulation of the input hpRNA molecules but no siRNAs were detectable (**Figures 1C, 2C**), indicating that that the full-length hpRNA was, if at all, only poorly processed by DCLs into few siRNAs below the detection limit of our method. In addition to the full-length hpRNA, RNA molecules smaller than full-length were detected, but these molecules were more likely hydrolytic degradation products rather than DCL-mediated processing products.

### Delivery of hpRNA by Petiole Absorption

**FIGURE 3:**
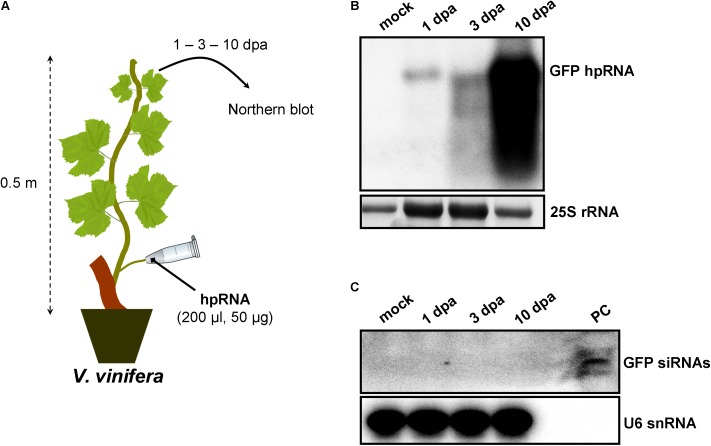
Delivery of exogenous hpRNA (500 nt) in *V. vinifera* by petiole absorption. **(A)** Apical leaves were harvested 1, 3, and 10 dpa and total RNA was extracted. **(B)** Northern blot for the detection of GFP hpRNA. Total RNA (5 μg) was analyzed in a 1.2% agarose/formaldehyde gel and the full-length GFP cDNA fragment (792 bp) was used as a hybridization probe. The ethidium bromide stained gel depicts the 25S rRNA band and serves as an RNA loading control. **(C)** Northern blot for the detection of GFP siRNAs. Total RNA (20 μg) was analyzed in a 15% polyacrylamide gel and the full-length GFP cDNA fragment (792 bp) was used as a hybridization probe. As positive control (PC) *in vitro* synthesized 22-nt GFP siRNAs (100 ng) were included. Hybridization of the membrane with a DNA oligo detecting the U6 snRNA served as an RNA loading control.

**FIGURE 4:**
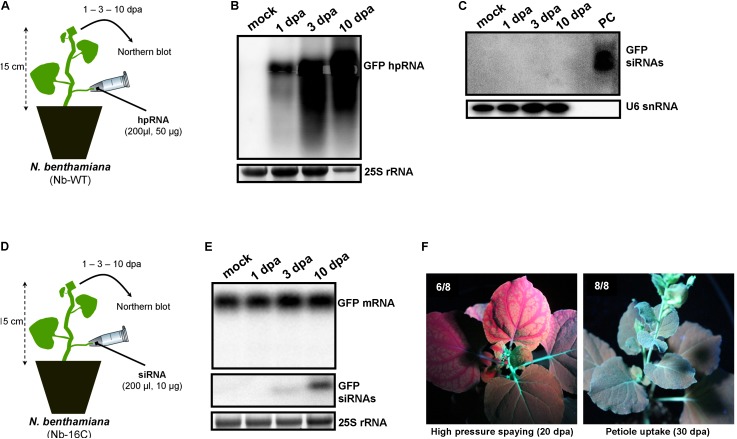
Delivery of exogenous hpRNA (500 nt) and siRNA (22 nt) in *N. benthamiana* wild type (Nb-WT) and GFP-expressing (Nb-16C) by petiole absorption. **(A)** Apical Nb-WT leaves were harvested 1, 3, and 10 dpa and total RNA was extracted. **(B)** Northern blot for the detection of GFP hpRNA in Nb-WT. Total RNA (5 μg) was analyzed in a 1.2% agarose/formaldehyde gel and the full-length GFP cDNA fragment (792 bp) was used as a hybridization probe. The ethidium bromide stained gel depicts the 25S rRNA band and serves as an RNA loading control. **(C)** Northern blot for the detection of GFP siRNAs in Nb-WT. Total RNA (20 μg) was analyzed in a 15% polyacrylamide gel and the full-length GFP cDNA fragment (792 bp) was used as a hybridization probe. As positive control (PC) *in vitro* synthesized 22-nt GFP siRNAs (100 ng) were included. Hybridization of the membrane with a DNA oligo detecting the U6 snRNA served as an RNA loading control. **(D)** Apical Nb-16C leaves were harvested 1, 3, and 10 dpa and total RNA was extracted. **(E)** Northern blot for the detection of GFP mRNA (upper panel) and GFP siRNAs (middle panel) in Nb-16C upon the petiole-mediated uptake of a 22-nt GFP siRNA. For the detection of GFP mRNA, total RNA (5 μg) was analyzed in a 1.2% agarose/formaldehyde gel and for the detection of GFP siRNAs total RNA (20 μg) was analyzed in a 15% polyacrylamide gel. The full-length GFP cDNA fragment (792 bp) was used as a hybridization probe. The ethidium bromide stained gel depicts the 25S rRNA band and serves as an RNA loading control. **(F)** Ultraviolet images of Nb-16C where the 22-nt GFP siRNA was delivered by high-pressure spraying (left panel) or petiole uptake (right panel). For each treatment, eight plants were monitored.

Since wounds generated by trunk drilling of RNA may offer entrance to pathogenic microorganisms, we sought to establish alternative and less abrasive methods for RNA delivery. We reasoned that, removing a leaf and applying a solution on the protruding cut stump of the petiole that is still attached to the plant, would lead to efficient uptake of the solution by the plant, most likely through capillary forces and vasculature flow. Indeed, by attaching tubes that contained hpRNA solutions to protruding petioles of *V. vinifera* (**Figure 3A**) and *N. benthamiana* (**Figure 4A**) revealed efficient uptake and transport of the applied hpRNA already 1 dpa (**Figures 3B, 4B**, respectively). In contrast to delivery of hpRNA by trunk drilling and injection, the hpRNA levels increased from day 1 up to 10 dpa (**Figures 3B, 4B**). This was a rather unexpected observation, given the fast vascular flow that normally occurs plants. It should be noted that the hpRNA solution was totally absorbed from the tube 1 dpa. Therefore, the gradual increase of hpRNA accumulation from 1 to 10 dpa was not due to the gradual uptake of the exogenously provided hpRNA, but rather reflected a peculiar physiological mechanism whose details are not very clear to us. Whatever the reasons underlying this observation may be, compared to trunk drilling, the petiole absorption method leads to slower but more long-lasting delivery of RNA molecules. Yet, similar to the trunk drilling method, the hpRNA was also largely intact and no siRNAs were detected when the petiole absorption method was applied (**Figures 3C, 4C**).

### Delivery of siRNAs by Petiole Absorption

**FIGURE 5:**
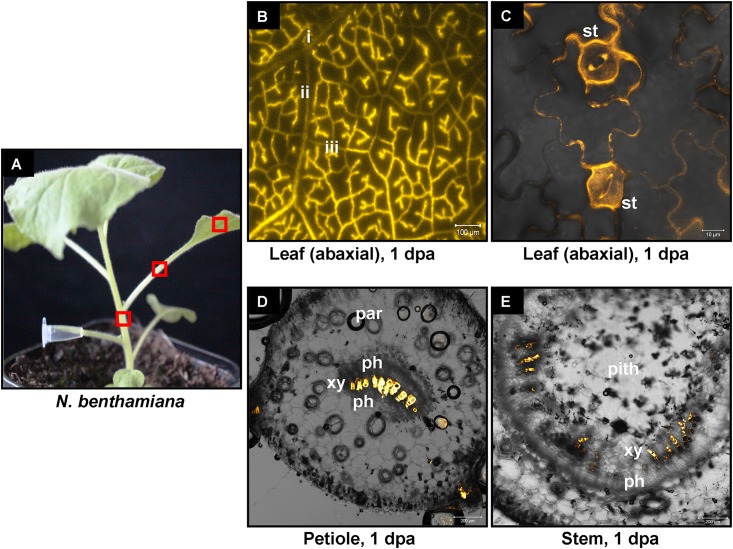
Delivery of CY3-labeled 22-nt siRNA by petiole absorption to *N. benthamiana.* **(A)** Red boxes indicate parts of the plant that were subjected to microscopy 1 dpa. **(B)** Fluorescence microscopy of the abaxial side of a distant leaf. **(C)** Confocal microscopy of the abaxial side of a distant leaf. **(D)** Confocal microscopy of the petiole (cross section) of a leaf. **(E)** Confocal microscopy of the stem (cross section) above the site of CY3-labeled siRNA application. Abbreviations: i, class I vein; ii, class II vein; iii, class III vein; st, stomata; xy, xylem; ph, phloem; par, parenchymatic cells.

**FIGURE 6:**
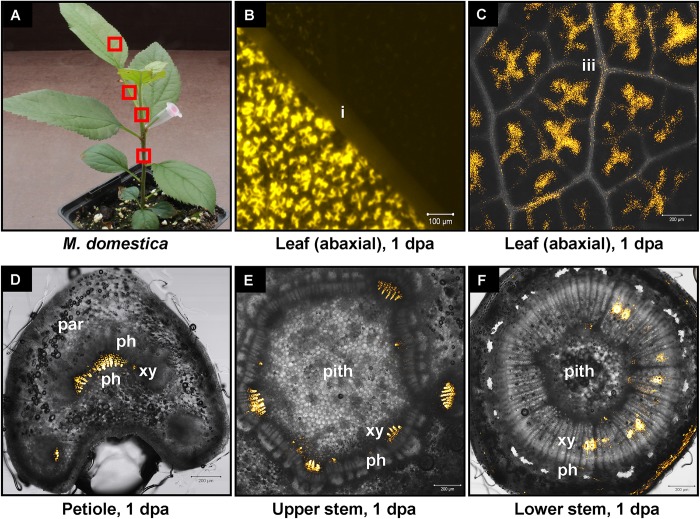
Delivery of CY3-labeled 22-nt siRNA by petiole absorption to young apple tree. **(A)** Red boxes indicate parts of the plant that were subjected to microscopy 1 dpa. **(B)** Fluorescence microscopy of the abaxial side of a distant leaf. **(C)** Confocal microscopy of the abaxial side of a leaf. **(D)** Confocal microscopy of the petiole (cross section) of a leaf. **(E)** Confocal microscopy of the stem (cross section) above the site of CY3-labeled siRNA application. **(F)** Confocal microscopy of the stem (cross section) below the site of CY3-labeled siRNA application. Abbreviations: i, class I vein; iii, class III vein; st, stomata; xy, xylem; ph, phloem; par, parenchymatic cells.

**FIGURE 7:**
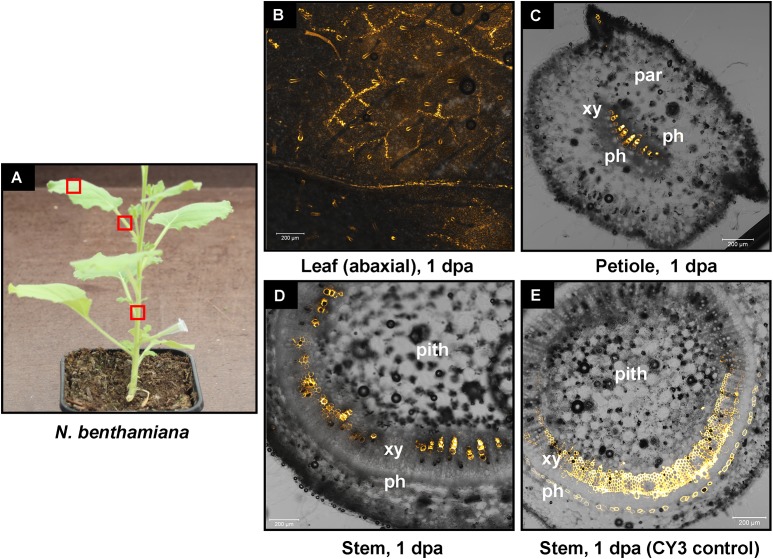
Delivery of CY3-labeled hpRNA by petiole absorption to *N. benthamiana*. **(A)** Red boxes indicate parts of the plant that were subjected to microscopy 1 dpa. **(B)** Confocal microscopy of the abaxial side of a leaf. **(C)** Confocal microscopy of the petiole (cross section) of a leaf. **(D)** Confocal microscopy of the stem (cross section) above the site of CY3-labeled hpRNA application. **(E)** Delivery of free CY3 dye (not conjugated to RNA molecule) by petiole absorption to *N. benthamiana*. Confocal microscopy of the stem (cross section) above the site of CY3 dye application. Abbreviations: xy, xylem; ph, phloem; par, parenchymatic cells.

We have previously shown that high pressure spraying of a 22-nt GFP siRNAs into GFP-expressing *N. benthamiana* (Nb-16C) (Voinnet and Baulcombe, 1997) triggers local and systemic silencing (Dalakouras et al., 2016). The same siRNA was applied by petiole absorption in Nb-16C (**Figure 4D**), and although efficiently uptaken, failed to target GFP mRNA for silencing (**Figure 4E**). Corroborating the Northern blot data, ultraviolet monitoring of Nb-16C plants that had uptaken the 22-nt GFP siRNA through their petioles exhibited no visible RNAi phenotype (**Figure 4F**, right panel). Taken together, the inability of hpRNA to be processed by DCLs into siRNAs and the failure of the delivered siRNAs to trigger silencing strongly suggested that the exogenously provided RNA (either hpRNA or siRNA) was not given access to the interior of the cell where these processes take place. In order to get detailed information about the transport and localization of the delivered RNA, an *in vitro* synthesized and HPLC-purified CY3-labeled 22-nt siRNA was applied by petiole absorption to *N. benthamiana* wild type plants (Nb-WT) (**Figure 5A**). Examination of the abaxial surface of a systemic leaf 1 dpa revealed the presence of the labeled siRNA in class I (midrib), class II, and class III veins (**Figure 5B**), according to the previously described vein classification system of *N. benthamiana* (Roberts et al., 1997). Confocal microscopy of the abaxial surface of leaves 1 dpa demonstrated that the transported RNA was restricted to the leaf apoplast and stomata guard cells (**Figure 5C**). Ultrastructural and histochemical studies on guard cells of various plant species revealed that stomata guard cells are symplastically isolated and lack plasmodesmata that would physically connect them with neighboring epidermal and mesophyll cells (Wille and Lucas, 1984). However, solutes are transported from the apoplast to mature guard cells (Wille and Lucas, 1984). Thus, the fact that we were able to detect the labeled siRNA in stomata guard cells strongly suggested an apoplastic route of the applied RNA molecule. Importantly, cross-section of the systemic petioles and stems clearly showed that the labeled siRNA was transported exclusively through the xylem and not the phloem (**Figures 5D,E**). Similar results suggesting exclusive xylem transport were obtained when the labeled siRNA was applied through petiole absorption in *M. domestica* plants (**Figure 6**), suggesting that there is virtually no difference in the transport of the exogenously delivered RNAs between herbaceous and woody plants. In order to test whether there are any differences between the transport of the siRNA and the much larger hpRNA, CY3-labeled hpRNA was applied through the petiole in *N. benthamiana* plants (**Figure 7A**). However, the hpRNA was also detected exclusively in the xylem (**Figures 7B–D**). In contrast, the free CY3 dye (control experiment) was detected in both xylem and phloem (**Figure 7E**), suggesting that, RNA molecules (siRNA or hpRNA) are too big to be transported into phloem cells. In general, phloem is the living tissue through which metabolites, but also RNA molecules, are transported throughout the plant according to a source-to-sink direction (Tournier et al., 2006). While phloem sieve elements lack nuclei and most probably DCL activity, DCLs are present and active in phloem companion cells, as verified by DCL-processing of companion cell-expressed dsRNAs in Arabidopsis (Dunoyer et al., 2005). In contrast, xylem mainly consists of non-living tracheids and vessel elements which are, to the best of our knowledge, devoid of DCL or any other RNAi activity, thus accounting for our observations. Whereas phloem flow is bidirectional, xylem flow is considered to be unidirectional, mainly mediating water transport from the roots to the upper parts of the plant. Yet, with our experimental setup we could show that xylem flow from leaf petioles to the rest of the plant can also take place.

### Perspectives for Pest Management

In insect cells, RNAi is generally initiated by cleavage of dsRNA by Dicer-2 into 21-nt siRNAs which are loaded onto the insect’s Ago-2 (Joga et al., 2016). Expressing in plants dsRNA may not be an optimal strategy to trigger RNAi of vital insect genes since it will be processed by plant DCLs into 21–22-nt siRNAs that will be loaded onto plant AGO1. It is likely that plant RISCs and plant-produced siRNAs inefficiently function in insects due to plant-specific biochemical modifications. Thus, delivery to plants of hpRNAs/dsRNAs that remain unprocessed by plant DCLs could be a suitable strategy to favor Dcl-2-mediated processing into siRNAs that would be functional in insects. Indeed, trans-kingdom RNAi against cotton wool worm (*Helicoverpa armigera*) was enhanced in an Arabidopsis *dcl2 dcl3 dcl4* triple mutant (Mao et al., 2007). Similarly, coleopteran insects (*Diabrotica* ssp.) were sensitive to RNAi upon feeding of dsRNAs but not siRNAs (Ivashuta et al., 2015). Importantly, Bally et al. (2016) observed a remarkable increase of insecticidal RNAi efficiency in transplastomic plants when dsRNAs were expressed in the plant chloroplasts which are devoid of DCLs. Delivery of dsRNAs into plants with methods different than the ones presented in this study (e.g., by mechanical inoculation or leaf spraying) resulted to their processing into siRNAs (Tenllado et al., 2003; Gan et al., 2010; Koch et al., 2016; Kaldis et al., 2017; Mitter et al., 2017; Niehl et al., 2018). In contrast, with the alternative methods described in this study, the delivered hpRNA was not processed by DCLs since it was mainly transported through the xylem and restricted to the apoplast, in both woody and herbaceous plants. As such, these methods could be potentially useful in pest management platforms against chewing insects or xylem sap-feeding insects and eukaryotic pathogens residing in the xylem.

## Materials and Methods

### Trunk Drilling

For *M. domestica*, 2 approximately 1.5 m tall plants with a 3 cm trunk diameter were used. With a conventional drill (Black and Decker) and a drill bit of 4 mm a hole was drilled into the stem approximately up to the tree’s pith. Into this hole, the hpRNA solution was gently applied (1 ml, 500 μg) using a 1 ml insulin syringe (without the needle). After the application the hole was not sealed and trees were grown in the glasshouse applying 25°C and a 16/8 h light/dark period. Sixteen pooled leaves (eight from each of the two plants) were harvested 1, 3, and 10 dpa and subjected to Northern blot analysis. The pooled material for each dpa was non-homogeneous and represented all possible leaf stages, from young and not fully expanded to mature and fully expanded leaves. For *V. vinifera*, three approximately 0.5 m tall plants with a 1.5 cm trunk diameter (rootstock) were used. Trunk drilling of the rootstock was performed as described above. Three pooled leaves (one from each of the three plants) were harvested 1, 3, and 10 dpa. The pooled material for each dpa was non-homogeneous and represented all possible leaf stages, from young not fully expanded to mature fully expanded leaves. Total RNA was extracted from the leaf material and subjected to Northern blot analysis.

### Petiole Absorption

For *V. vinifera*, three approximately 0.5 m tall plants were used. The first leaf was detached and a 0.5 ml tube whose bottom was cut off and which contained the hpRNA solution (200 μl, 50 μg) was attached to the protruding petiole. Three leaves (one from each of the three plants) were harvested and pooled 1, 3, and 10 dpa, respectively. The pooled material for each dpa was non-homogeneous and represented all possible leaf stages, from young not fully expanded to mature fully expanded leaves (e.g., a young not expanded leaf from plant 1, an almost fully expanded leaf from plant 2, and a fully expanded leaf from plant 3 were pooled together and used for the 1 dpa analysis, etc). Only leaves above the site of RNA application were sampled. Total RNA was extracted from the leaf material and subjected to Northern blot analysis. For *N. benthamiana*, six approximately 10 cm tall plants were used. RNA delivery was performed as described above. Six leaves (one from each of the six plants) were harvested and pooled 1, 3, and 10 dpa, respectively. The pooled material for each dpa was non-homogeneous and represented different leaf stages, from young not yet expanded to almost fully expanded leaves (at that stage the plants were still quite young and fully expanded leaves were not yet available). Only leaves above the site of RNA application were sampled. Total RNA was extracted from the leaf material and subjected to Northern blot analysis.

### High Pressure Spraying

One hundred microliters of an aqueous siRNA solution (8 μM) was sprayed from a 2–4 cm distance at the abaxial surface of leaves with the CONRAD air brush gun AFC-250A ^1^ and at a pressure of 7–8 bar provided by the METABO Elektra Beckum Classic 250 compressor ^2^.

### Northern Blot Analysis

Total RNA was extracted with Spectrum Plant Total RNA Kit^3^ according to the manufacturer’s instruction. For Northern blot analysis of large RNA molecules, 5 μg of total RNA was separated on a 1.2% agarose formaldehyde gel, capillary transferred onto the positively charged nylon membrane Bright Star^4^ and UV_312nm_-cross-linked (300 mJ/cm^2^). For the detection of GFP hpRNA and GFP mRNA, the PerfectHyb Plus 1x (see footnote 3) was used for overnight hybridization at 64°C with the random-primed αα-^32^P-dCTP labeled GFP cDNA fragment (792 bp). Membranes were washed at 64°C with buffer 1 [2× SSC, 0.1% SDS (w/v)] for 30 min and with buffer 2 [0.5× SSC, 0.1% SDS (w/v)] for 15 min. Membranes were then exposed to FujiFilm Imaging Plates^5^ for 24 h and scanned using PharosFX Plus PhosphorImager^6^. For Northern blot analysis of small RNA molecules, 20 μg were separated on a 15% TBE-urea gel^7^ at 120 V for 4 h. Gels were blotted onto the positively charged nylon membranes Bright Star (see footnote 4) by electro-blotting at 300 mA for 1 h and membranes were UV_312nm_-cross-linked (300 mJ/cm^2^). For detection of GFP siRNAs, the random-primed α-^32^P-dCTP labeled GFP cDNA fragment (792 bp) was used as a hybridization probe. For detection of U6 snRNA, the DNA oligo 5′-AGG GGC CAT GCT AAT CTT CTC-3′ was used in an end-labeling reaction using γ-^32^P ATP^8^ and T4 polynucleotide kinase^9^ as a hybridization probe. The hybridization temperature was 42°C and membranes were washed twice with buffer 1 [2× SSC, 0.1% SDS (w/v)] at 42°C for 30 min.

### Generation of hpRNA and siRNA

The 500-nt *in vitro* transcribed GFP hpRNA was provided by BASF (Limburgerhof). The CY3-labeling of the hpRNA was performed with the Silencer siRNA labeling kit^10^ according to the manufacturer’s instructions. The 22-nt siRNA was *in vitro* synthesized by Metabion^11^. The HPLC-purified CY3-labeled 22-nt siRNA was *in vitro* synthesized by Sigma-Aldrich (see footnote 3).

### Microscopy Imaging

For confocal laser microscopy, sections were imaged using a Zeiss LSM 510 confocal laser setup with an Axio Observer Z1 inverted microscope. Cy3 dye was excited at 514 nm with a diode pumped solid state laser and its fluorescence was detected using a 575–615 nm red bandpass filter. Overview images of entire stem and root sections were taken with a Zeiss FLUAR 5x objective, while for detailed images of leaf cells we used a 40x Zeiss C-Apochromat with water immersion. For fluorescence stereomicroscopy, the abaxial surface of a leaf was imaged under CY3 filter with a Zeiss SteREO Lumar.V12 stereomicroscope with a 0.8× objective at 80× magnification.

## Author Contributions

AD, GK, and MW conceived the experiments. AD, WJ, GB, AB, MB, and TM performed the experiments. AD and MW wrote the manuscript.

## Conflict of Interest Statement

The authors declare that the research was conducted in the absence of any commercial or financial relationships that could be construed as a potential conflict of interest.

## Footnotes

1 www.conrad.de

2 www.metabo.com
